# Investigating Blood Donors With Postdonation Respiratory Tract Symptoms During the Wild-Type, Delta, and Omicron Waves of the Coronavirus Disease 2019 Pandemic in England

**DOI:** 10.1093/ofid/ofad499

**Published:** 2023-10-05

**Authors:** Shannah Gates, Samreen Ijaz, Hatice Baklan, Charlotte Washington, Su Brailsford, Maria Zambon, Heli Harvala

**Affiliations:** Microbiology Services, NHS Blood and Transplant, London, United Kingdom; Virus Reference Department, United Kingdom Health Security Agency, London, United Kingdom; Microbiology Services, NHS Blood and Transplant, London, United Kingdom; Microbiology Services, NHS Blood and Transplant, London, United Kingdom; Microbiology Services, NHS Blood and Transplant, London, United Kingdom; Virus Reference Department, United Kingdom Health Security Agency, London, United Kingdom; Microbiology Services, NHS Blood and Transplant, London, United Kingdom; Radcliffe Department of Medicine, University of Oxford, Oxford, United Kingdom

**Keywords:** post-donation information, SARS-CoV-2 RNA, SARS-CoV-2 variants

## Abstract

**Background:**

Severe acute respiratory syndrome coronavirus 2 (SARS-CoV-2) infection has been shown to be detectable in blood from infected individuals. Though RNAemia frequencies are typically low, the presence of potentially infectious virus potentially poses a transmission risk during blood transfusion.

**Methods:**

Archived plasma samples were collected from blood donors who later reported possible SARS-CoV-2 infection with the wild-type strain, Delta variant, or Omicron variant. This was based on either symptom onset or a positive test within 2 weeks from their donation. Donations were tested for SARS-CoV-2 RNA, and information on symptoms and testing results were gathered during postdonation interview.

**Results:**

Of 518 archived plasma samples tested, 19 (3.7%) were found to have detectable levels of SARS-CoV-2 RNA. SARS-CoV-2 RNA was detected in donors who donated during the Delta (10/141 [7.1%]) and Omicron (9/162 [5.6%]) waves. SARS-CoV-2 RNA was not detected in donors who donated during the wild-type wave (0/215). Seventeen of 19 RNAemic donors reported symptom onset or a positive test within 2 days of donating. SARS-CoV-2 RNA was detected in asymptomatic or presymptomatic blood donors.

**Conclusions:**

Despite RNAemia being correlated with SARS-CoV-2 disease severity, RNAemia was detected in asymptomatic or presymptomatic blood donors.

Severe acute respiratory syndrome coronavirus 2 (SARS-CoV-2) emerged in the Hubei province of China in late 2019, as a cause of respiratory disease occasionally leading to acute respiratory distress syndrome and death [1]. Increasing age, male sex, smoking, and comorbidities such as cardiac disease, hypertension, and diabetes have been identified as risk factors for severe infections (reviewed in [2]). Since then, >20 million SARS-CoV-2 cases have been diagnosed in the United Kingdom (UK), leading to >150 000 deaths [3].

The initial surge of cases was caused by the wild-type strain of SARS-CoV-2, whereas the more infectious strains of SARS-CoV-2, named as Alpha, Delta, and Omicron variants, were responsible for rise in the infection rates during winter 2020–2021, summer 2021, and winter 2021–2022 [3]. Although SARS-CoV-2 shedding in plasma or serum is uncommon (<10%) and was initially largely associated with severe infections only [2], no data are available for RNAemia associated with particular SARS-CoV-2 variants. Although the transfusion-associated transmission of SARS-CoV-2 remains possible, no cases of transfusion-transmitted SARS-CoV-2 infection have been reported to date.

As a safety precautionary measure, donors are asked to contact blood services if they experience any postdonation respiratory symptoms within 5 days from their donation. This would lead to all blood components manufactured from the donation to be discarded. The potential impact of these discards to the blood supply during periods of high rates of SARS-CoV-2 infections and whether this precautionary step is mandated led us to investigate frequencies of RNAemia by different SARS-CoV-2 variants in such donors and their potential transmission risk and whether the current 5-day discard period for could be shortened.

## METHODS

### Study Population

Data on 518 blood donors reporting postdonation information relating to a possible SARS-CoV-2 infection with the wild-type strain (from 3 March to 11 June 2020), Delta variant (5 July to 13 October 2021), and Omicron variant (22 December 2021 to 31 December 2021) were included in this study. Those with either symptom onset or a positive test within 2 weeks from their donation were included in this study. Data collected included date of donation; reason for postdonation information, including onset and nature of symptoms and, if any, positive antigen or reverse-transcription polymerase chain reaction (RT-PCR) test; date of diagnosis; and if hospitalization was required. Further characteristics including age, sex, and ethnicity were obtained for donors whose donation was SARS-CoV-2 RNA positive.

### Patient Consent Statement

Ethical approval for the study and design of the work was given by the NHS Blood and Transplant (NHSBT) Blood Supply Clinical Audit, Risk and Effectiveness Committee research subgroup committee (reference number BSCR20010).

### Detection of SARS-CoV-2 RNA in Archived Plasma Samples of Those Reporting COVID-19–Related Postdonation Information

Archive plasma samples were tested for SARS-CoV-2 RNA; samples collected during the first wave were tested at the UK Health Security Agency as previously reported [4]. They were tested using an RT-PCR assay targeting the RdRp gene with a sensitivity of 3.7 copies/reaction. Archive plasma samples collected during the Delta and Omicron wave were tested at the NHSBT Microbiology Services Laboratory. NHSBT Microbiology Services Laboratory detected SARS-CoV-2 RNA by transcription-mediated amplification using the Procleix SARS-CoV-2 Assay (Grifols) performed on the Panther automated system (Grifols Diagnostic Solutions) according to the manufacturer's instructions for use. A SARS-CoV-2–positive control sample was included on all runs for quality control purposes. The 95% limit of detection for this assay in plasma is stated in the instructions for use as 9.19 copies/mL. Due to small sample volumes (<900 μL), no further confirmatory testing was possible.

## RESULTS

A total of 518 blood donors with COVID-19–related postdonation information were included in this study. From these, 215 reported postdonation information during the first pandemic wave when most infections were caused by the wild-type SARS-CoV-2 strain in the UK. Due to limited testing availability at this time, respiratory samples from only 12 donors were confirmed positive for SARS-CoV-2. Most of these donors, however, reported symptoms consistent with SARS-CoV-2 (n = 211). For the majority of donors, symptom onset or a positive test result was reported within 4 days of donation (n = 184 [85.6%]; Table 1). No SARS-CoV-2 RNA was detected in any of the plasma samples tested from these donors (Table 1).

**Table 1.** Detection of Severe Acute Respiratory Syndrome Coronavirus 2 RNA in Blood Donors Reporting Postdonation Symptoms or a Positive Test During the First Pandemic Wave and Delta and Omicron Variant Waves

Symptom Onset (Days After Donation)	Wild-Type SARS-CoV-2	Delta Variant		Omicron Variant	
Same day	0/26	1/15	(6.7%)	2/17	(11.8%)
1 d	0/39	5/27	(14.8%)	5/38	(13.2%)
2 d	0/64	4/35	(11.4%)	1^a^/40	(2.5%)
3 d	0/32	0/16	…	0/33	…
4 d	0/23	0/21	…	1/18	(5.6%)
≥5 d	0/31	1^a^/27	(3.7%)	0/16	…
Total	0/215	10/141	(7.1%)	9/161	(5.6%)

Abbreviation: SARS-CoV-2, severe acute respiratory syndrome coronavirus 2.

^a^Donor asymptomatic at time of reporting postdonation information and test positivity used instead of symptom onset postdonation.

An additional 141 blood donors reporting COVID-19–related postdonation information during the Delta wave were similarly investigated. The majority of these donors reported either a positive RT-PCR (n = 80) and/or lateral flow (n = 48) result. Most also had symptoms consistent with SARS-CoV-2 (n = 124). Symptom onset or a positive test result was within 4 days of donation for the majority of donors (n = 114 [80.5%]; Table 1). From these donors, a total of 10 archive samples tested positive for SARS-CoV-2 RNA (10/141 [7.09%]); these were mostly (9/10) from donors with symptom onset or a positive test within 2 days of their donation (Table 1).

This study was repeated in the beginning of the Omicron wave with data collected for 162 blood donors with COVID-19–related postdonation information over a 23-day period. The majority of these donors reported either a positive RT-PCR (n = 50) and/or lateral flow test (n = 113) result and/or symptoms consistent with SARS-CoV-2 infection (n = 124). SARS-CoV-2 RNA was detected in 9 archived plasma samples (9/162 [5.6%]; Table 1). Similar to the Delta wave, these were mostly obtained from donors with symptom onset or a positive test within 2 days of their donation (8/9; Table 1).

All 19 SARS-CoV-2 RNA–positive donations were from donors who had subsequently tested positive for SARS-CoV-2 either by RT-PCR or by lateral flow test. Of these, 13 donors had only mild symptoms at the time of reporting postdonation information, whereas 2 donors were completely asymptomatic 2 days and 6 days postdonation (Table 2). Mean age of donors with detectable SARS-CoV-2 RNA in their blood was 52 years, and approximately one-half were males (11/19 [58%]). The average age of donors with detectable SARS-CoV-2 RNA in their blood was lower during the Delta wave than the Omicron wave (mean age, 46 years vs 59 years; Table 2). None of the SARS-CoV-2 RNA–positive donations were supplied for clinical use.

**Table 2.** Description of Donors With Severe Acute Respiratory Syndrome Coronavirus 2 RNA Detected in Plasma During the Delta Variant Wave, July–October 2021 (n = 10), and During the Beginning of the Omicron Variant Wave, December 2021 (n = 9)

Variant Case Number	Sex	Age Group, y	Ethnicity	Date of Donation	Method of SARS-CoV-2 Diagnosis	Symptoms Reported	Days Between Onset and Donation
Delta 1	Female	31–40	White British	20 Jul 2021	PCR	Yes	2
Delta 2	Male	51–60	Asian	5 Aug 2021	PCR	Yes	2
Delta 3	Female	41–50	White British	30 Aug 2021	Antigen	Yes	1
Delta 4	Female	31–40	Mixed Caribbean	3 Sep 2021	PCR	Yes	2
Delta 5	Male	41–50	White British	3 Sep 2021	PCR	Yes	2
Delta 6	Male	51–60	White British	6 Sep 2021	PCR	Yes	1
Delta 7	Male	21–30	White British	21 Sep 2021	Antigen	Yes	1
Delta 8	Female	41–50	White British	23 Sep 2021	PCR	No	6
Delta 9	Male	61–70	White British	8 Oct 2021	Antigen	Yes	3
Delta 10	Male	51–60	White British	13 Oct 2021	No test	Yes	1
Omicron 1	Male	61–70	White British	24 Dec 2021	Antigen	Yes	1
Omicron 2	Female	21–30	White British	20 Dec 2021	Antigen	Yes	1
Omicron 3	Male	41–50	White British	27 Dec 2021	Antigen	Yes	0
Omicron 4	Male	71–80	White British	29 Dec 2021	Antigen	Yes	1
Omicron 5	Female	61–70	White British	26 Dec 2021	PCR	Yes	1
Omicron 6	Female	71–80	White British	24 Dec 2021	PCR	Yes	4
Omicron 7	Male	51–60	White British	30 Dec 2021	Antigen	Yes	1
Omicron 8	Female	41–50	White British	31 Dec 2021	Antigen	Yes	0
Omicron 9	Male	71–80	White British	29 Dec 2021	Antigen	No	2

Abbreviations: PCR, polymerase chain reaction; SARS-CoV-2, severe acute respiratory syndrome coronavirus 2.

## DISCUSSION

This study found evidence of SARS-CoV-2 RNA in 19 of 518 (3.7%) blood donations studied, noting that none of the donors with suspected wild-type SARS-CoV-2 infection had detectable RNA. Ten of the 19 cases likely relate to donor infection with the Delta variant, which circulated during the third wave, and the remaining 9 cases with the Omicron variant. Most donors with suspected Delta and Omicron infections had tested positive for SARS-CoV-2 either by RT-PCR or antigen test (121/144 and 112/149, respectively). It is noteworthy that testing capacity was limited during the wild-type wave; therefore, only 12 of 218 donors reported being tested for SARS-CoV-2. Without a positive test result, it is possible that some of those tested during the first wave actually had another respiratory virus and not SARS-CoV-2. Due to testing limitations, samples obtained from donors with suspected wild-type SARS-CoV-2 infection were run on different assays at different sites compared to the Omicron and Delta donor samples. Therefore, the lack of detection of SARS-CoV-2 RNA during the wild-type wave could also be due to differences in sensitivity of the RT-PCR assays. It is also possible that Delta and Omicron variants do cause more pronounced RNAemia than the wild-type SARS-CoV-2 strain, hence the greater detection rates.

A slightly smaller proportion of donors had detectable SARS-CoV-2 RNAemia during the Omicron wave versus the Delta wave (7.1% vs 5.6%). This effect could be due to increased vaccination rates during the Omicron wave versus the Delta wave (81% vs 59%). In the United States, it was found in blood donors that there was a reduction in RNA detection in plasma that coincided with the timing of mass vaccination across the country [5].

Several studies to date have shown a correlation between RNAemia and severity of SARS-CoV-2 infection, with higher levels being linked to mortality [6–10]. We detected RNAemia in 4 asymptomatic cases, from which 2 reported symptoms 4 and 5 days after their donation. Therefore, our finding shows that RNAemia is not only found in persons who are severely ill but can be present both before symptom onset and in those who are asymptomatic. Similar studies in other countries have also found detectable SARS-CoV-2 RNA in presymptomatic and asymptomatic blood donors. A French study found that 3.4% of 1092 blood donors reporting COVID-19–related postdonation information had detectable SARS-CoV-2 RNAemia [11]. A study in the United States found that 8.7% of 2250 blood donors reporting COVID-19–related information postdonation had detectable SARS-CoV-2 RNAemia, although they found this reduced to 4% after widespread vaccination [5]. The mechanism by which SARS-CoV-2 enters the bloodstream, especially in presymptomatic or asymptomatic people, is unknown. The finding of RNAemia prior to symptom onset suggests that systemic infection may play a role in COVID-19, although the clinical significance of this remains undetermined.

Whether detection of SARS-CoV-2 RNA in blood directly equates to (infectious) viremia remains controversial. A recent study demonstrated that intact SARS-CoV-2 virions were present in plasma pellets of those infected [8]. The finding of RNAemia in blood donors, although in a small percentage, is a concern to blood safety as it suggests a possible risk of transfusion transmission. However, despite high levels of infection worldwide, there has so far been no confirmed case of transfusion-transmitted SARS-CoV-2 infection. In addition to this, several studies have also attempted to culture infectious SARS-CoV-2 from plasma, but none has succeeded [5, 11, 12]. Furthermore, inoculation of highly susceptible mice intravenously with plasma containing SARS-CoV-2 RNA failed to transmit infection [5]. These studies suggest that the virus is not viable in plasma, and is thus not likely to be transmitted by transfusion. Italy has recently removed the requirement to discard blood products from donors who test positive or report symptoms of SARS-CoV-2 after donation [13].

In our study during the Delta and Omicron waves, the frequency of RNA detection was highest among those who developed symptoms between 1 and 2 days after their donation (10.7% [15/140]), but only 2 of 84 donors reporting were shown to harbor SARS-CoV-2 RNA if they developed symptoms or tested positive >4 days after the donations (2.4%; *P* < .05 by χ^2^ test) (Table 3).

**Table 3.** Presence of Severe Acute Respiratory Syndrome Coronavirus 2 RNA in Plasma in Relation to Donor Testing Results and Presence of Symptoms During the Delta and Omicron Waves

Testing and Symptoms	RNA Detected in Plasma	RNA Not Detected in Plasma	Percentage RNA Positive
SARS-CoV-2 testing^a^ and symptoms			
SARS-CoV-2 negative with symptoms	0	35	0.0%
SARS-CoV-2 positive with symptoms	17	230	6.9%
SARS-CoV-2 positive without symptoms	2	22	8.3%
Onset of symptoms			
Same day as donation	0	29	0.0%
1 d after donation	9	65	12.2%
2 d after donation	6	75	7.4%
3 d after donation	0	53	0.0%
4 d after donation	1	35	2.8%
≥5 d after donation	1	49	2.0%

Abbreviation: SARS-CoV-2, severe acute respiratory syndrome coronavirus 2.

^a^SARS-CoV-2 testing of nasopharyngeal samples by reverse-transcription polymerase chain reaction or antigen assay.

These data, together with the greatly increased SARS-CoV-2 case numbers reported following the emergence of the more transmissible Omicron variant, motivated us to reevaluate our response to postdonation information about possible or confirmed SARS-CoV-2 infection. The number of postdonation information calls with likely or confirmed SARS-CoV-2 infection increased from around 4 per week during the Delta wave to up to 23 a day during the early Omicron wave in December 2021 in England (Supplementary Table 1). These indicate the substantial impact on discards within 5 days of donation and exacerbate concerns for red cell unit stock levels. As we demonstrated, the frequency of RNAemia was low in blood donors who had developed symptoms consistent with SARS-CoV-2 >2 days after donations; hence, it may be appropriate to reduce the discard interval frequency from 5 to 2 days. The early 2020 European Centre for Disease Prevention and Control guidance recommended the discard of components if confirmed or probable SARS-CoV-2 infection developed within 72 hours of donation, considering the largely theoretical risk of transmission via blood transfusion. Ongoing monitoring, however, is needed to measure its effect in reducing blood component waste, while at the same time being alert to and actively investigating any potential occurrences of transfusion-transmitted infections by this route. This study shows that hemovigilance is key to managing responses to threats to blood safety and supply.

## Supplementary Material

ofad499_Supplementary_Data

## Supplementary Data

Supplementary materials are available at *Open Forum Infectious Diseases* online. Consisting of data provided by the authors to benefit the reader, the posted materials are not copyedited and are the sole responsibility of the authors, so questions or comments should be addressed to the corresponding author.

## Notes

***Financial support.*** This work was supported by the National Institute for Health and Care Research (grant number NIHR203338 to H. H. and S. G.).

## References

[1] Zhu N , Zhang D, Wang W, et al A novel coronavirus from patients with pneumonia in China, 2019. N Engl J Med 2020; 382:727–33. 31978945 10.1056/NEJMoa2001017 PMC7092803

[2] Gao YD , Ding M, Dong X, et al Risk factors for severe and critically ill COVID-19 patients: a review. Allergy 2021; 76:428–55. 33185910 10.1111/all.14657

[3] UK Health Security Agency . Coronavirus (COVID-19) in the UK: England summary. 5 September 2023.

[4] Corman VM , Landt O, Kaiser M, et al Detection of 2019 novel coronavirus (2019-nCoV) by real-time RT-PCR. Euro Surveill 2020; 25:2000045. 10.2807/1560-7917.ES.2020.25.3.2000045 PMC6988269 31992387

[5] Saá P , Fink RV, Bakkour S, et al Frequent detection but lack of infectivity of SARS-CoV-2 RNA in presymptomatic, infected blood donor plasma. J Clin Invest 2022; 132:e159876. 10.1172/JCI159876 PMC9435642 35834347

[6] Hagman K , Hedenstierna M, Gille-Johnson P, et al Severe acute respiratory syndrome coronavirus 2 RNA in serum as predictor of severe outcome in coronavirus disease 2019: a retrospective cohort study. Clin Infect Dis 2021; 73:e2995–3001. 32856036 10.1093/cid/ciaa1285 PMC7499508

[7] Hogan CA , Stevens BA, Sahoo MK, et al High frequency of SARS-CoV-2 RNAemia and association with severe disease. Clin Infect Dis 2021; 72:e291–5. 32965474 10.1093/cid/ciaa1054 PMC7543277

[8] Jacobs JL , Bain W, Naqvi A, et al Severe acute respiratory syndrome coronavirus 2 viremia is associated with coronavirus disease 2019 severity and predicts clinical outcomes. Clin Infect Dis 2022; 74:1525–33. 34374761 10.1093/cid/ciab686 PMC9070832

[9] Li Y , Schneider AM, Mehta A, et al SARS-CoV-2 viremia is associated with distinct proteomic pathways and predicts COVID-19 outcomes. J Clin Invest 2021; 131:e148635. 10.1172/JCI148635 PMC8245177 34196300

[10] Prebensen C , Myhre PL, Jonassen C, et al Severe acute respiratory syndrome coronavirus 2 RNA in plasma is associated with intensive care unit admission and mortality in patients hospitalized with coronavirus disease 2019. Clin Infect Dis 2021; 73:e799–802. 32888003 10.1093/cid/ciaa1338 PMC7499533

[11] Cappy P , Candotti D, Sauvage V, et al No evidence of SARS-CoV-2 transfusion transmission despite RNA detection in blood donors showing symptoms after donation. Blood 2020; 136:1888–91. 32871595 10.1182/blood.2020008230 PMC7568032

[12] Andersson MI , Arancibia-Carcamo CV, Auckland K, et al SARS-CoV-2 RNA detected in blood products from patients with COVID-19 is not associated with infectious virus. Wellcome Open Res 2020; 5:181. 33283055 10.12688/wellcomeopenres.16002.1 PMC7689603

[13] Focosi D , Franchini M. SARS-Cov-2 and the safety of blood donations: time for a brave revision? Transfusion 2022; 62:717–9. 35128657 10.1111/trf.16818 PMC9115331

