# Supplementary material for: Investigating Blood Donors With Postdonation Respiratory Tract Symptoms During the Wild-Type, Delta, and Omicron Waves of the Coronavirus Disease 2019 Pandemic in England
Source: Open Forum Infect Dis. 2023 Oct 5;10(10):ofad499. doi: 10.1093/ofid/ofad499 (PMC10590102; doi:10.1093/ofid/ofad499)
Supplement: ofad499_Supplementary_Data [file ofad499_supplementary_data.docx]

**SUPPLEMENTARY DATA/ FOR INFORMATION ONLY**

**Supplemental Table 1.** Number of covid related PDI calls during different variant waves

| **Variant** | **Number of Covid PDIs** | **Time period/ days** | **Average calls per day** |
| --- | --- | --- | --- |
| *Wild type* | 350 | 100 | 4 |
| *Delta* | 382 | 102 | 4 |
| *Omicron* | 204 | 14 | 15 |
